# Booster Immunisation with Skin-Patch-Delivered Unadjuvanted SARS-CoV-2 Spike Protein Vaccine Is Safe and Immunogenic in Healthy Adults

**DOI:** 10.3390/vaccines14010028

**Published:** 2025-12-25

**Authors:** Christopher L. D. McMillan, David A. Muller, Germain J. P. Fernando, Alexandra C. I. Depelsenaire, Cesar Jayashi-Flores, Kelly-Anne Masterman, Sarika Namjoshi, Kartik Vyas, Deborah Pascoe, Julian Hickling, Stephanie Wallace, Daniel Duijsings, Joelle Vink, Adam K. Wheatley, Jennifer Juno, Greg Siller, Angus H. Forster

**Affiliations:** 1 School of Chemistry and Molecular Biosciences, The University of Queensland, St. Lucia, Brisbane, QLD 4072, Australia; c.mcmillan1@uq.edu.au (C.L.D.M.); brisbane456@gmail.com (G.J.P.F.); sdepelsenaire@vaxxas.com (A.C.I.D.); 2 Australian Infectious Diseases Research Centre, Brisbane, QLD 4072, Australia; 3 Global Virus Network Centre of Excellence, Brisbane, QLD 4029, Australia; 4 Vaxxas PTY Ltd., Hamilton, QLD 4007, Australia; cjayashi@une.edu.au (C.J.-F.); kamasterman@vaxxas.com (K.-A.M.); snamjoshi@vaxxas.com (S.N.); kvyas@vaxxas.com (K.V.); dpascoe@vaxxas.com (D.P.); 5 Working in Tandem Ltd., 23 Rathmore Road, Cambridge CB1 7AB, UK; julian@workingintandem.co.uk; 6 University of the Sunshine Coast Clinical Trials Centre, Sippy Downs, QLD 4556, Australia; swallac1@usc.edu.au; 7 Cerba Research, Marconistraat 16, 3029 AK Rotterdam, The Netherlandsjoelle.vink@cerbaresearch.com (J.V.); 8 Department of Microbiology and Immunology, The Peter Doherty Institute for Infection and Immunity, University of Melbourne, Melbourne, VIC 3000, Australia; a.wheatley@unimelb.edu.au (A.K.W.); jennifer.juno@unimelb.edu.au (J.J.); 9 Siller Medical, The University of Queensland, St. Lucia, Brisbane, QLD 4072, Australia; sillermedical@cbderm.au

**Keywords:** microarray patch, microneedles, SARS-CoV-2, HexaPro, phase 1 clinical trial, thermostable vaccine

## Abstract

Background/Objective: Despite available SARS-CoV-2 vaccines, coverage gaps persist due to unequal distribution and limited access. Microarray patches offer a promising solution to address these challenges, providing a safer and easier-to-use alternative. We present a randomised, double-blind Phase I clinical trial evaluating the SARS-CoV-2 spike protein subunit vaccine, HexaPro, delivered via a high-density microarray patch (HD-MAP). Methods: Forty-four healthy adults aged 18–50 years were assigned to receive either 0 µg, 15 µg, or 45 µg of HexaPro via the HD-MAP, with the primary objective of assessing safety and tolerability. Results: The HD-MAP HexaPro vaccine was found to be safe and well tolerated, with only mild adverse events reported. Following vaccination significant increases in spike-specific IgG titers were observed by 7 days and remained stable through day 90. This IgG response effectively neutralised multiple SARS-CoV-2 variants. Additionally, the HexaPro HD-MAP was stable for up to 12 months at 40 °C. Conclusions: These findings support the continued clinical development of HD-MAPs as an alternative vaccination strategy.

## 1. Introduction

While effective SARS-CoV-2 vaccines are currently available, there exists significant vaccination coverage discrepancies globally, especially in low- and middle-income countries (LMICs). Although many countries have stopped reporting coverage rates, making conclusions difficult, figures from 2023 indicate that, whilst 70% of the global population had received at least a single COVID-19 vaccine dose, those numbers drop to just 6.4% in some countries in Africa [1]. In Papua New Guinea, as of October 2023, just 4.2% of the population had received at least one dose [2]. Therefore, combined with the ongoing circulation and emergence of new viral variants, there exists a clear need for continued vaccination campaigns, with alternative vaccines and vaccination strategies to ensure equitable access [3]. Microarray patches (MAPs) represent one such alternative strategy to address these issues, and several different vaccines have been evaluated in clinical trials, including influenza [4,5,6,7], measles rubella [8,9], and Japanese encephalitis [10]. MAPs offer a multitude of advantages over traditional needle-and-syringe injection techniques, including ease of use, thereby negating the need for highly trained healthcare workers, ensuring safety with no sharps waste production, no requirement for reconstitution or multi-dose vials, simplified logistics and distribution, and increased thermostability [11,12,13,14].

One such MAP being developed for commercial use is the Vaxxas high-density MAP (HD-MAP). The HD-MAP is unique in the field in that it contains thousands of microprojections per square centimeter. Made of medical-grade polymer, the vaccine solution payload is deposited directly onto the tips of the projections where it rapidly dries. The HD-MAP is then applied to the skin at ~20–25 m/s using a custom auto-disabling applicator device. This dynamic application allows the microneedles to penetrate the skin to deliver the vaccine to the immune cell-rich epidermis and upper dermis layers of the skin while simultaneously causing localised cell death, serving as a physical immune enhancer [15], reducing or removing the need for a chemical adjuvant. Indeed, delivery of vaccines via the HD-MAP has routinely shown enhanced immunogenicity profiles in both preclinical and clinical studies, compared to needle-and-syringe delivery, for a wide variety of vaccine types [4,9]. Furthermore, by adding stabilising excipients, vaccines coated on the HD-MAP exhibit impressive thermostability profiles, even at elevated temperatures [4,16,17,18,19,20,21,22]. This has implications for last-mile delivery of vaccines to remote areas where cold chain storage infrastructure is lacking. The auto-disabling applicator is very easy to use, allowing potential self-administration. It is single-use and only requires the protective foil to be peeled back before application to the skin. Depressing the back of the applicator results in firing of the spring-loaded mechanism, which applies the HD-MAP to the skin. Combined, the HD-MAP makes an ideal platform for vaccination both in high-income countries (HICs) and low- and middle-income countries (LMIC) [23,24,25,26,27,28,29,30].

We have previously performed preclinical studies on the delivery of a recombinant SARS-CoV-2 spike protein vaccine candidate, HexaPro, via the HD-MAP [31]. HexaPro is a recombinant SARS-CoV-2 spike protein vaccine candidate (matching the spike sequence of early virus isolates) containing six stabilising proline insertions and a modified furin cleavage site that serve to increase the stability of the protein [32]. HexaPro has been shown to be an effective vaccine candidate when delivered in multiple forms in preclinical studies, including via the HD-MAP [31,33,34] and also in clinical trials via needle-and-syringe injection as an inactivated Newcastle disease virus-based vaccine [35,36]. We previously demonstrated that a HexaPro HD-MAP vaccination regime induced potent neutralising antibodies and cellular immune responses, which resulted in complete protection from infection in mouse models even in the absence of an adjuvant [31]. Additionally, the addition of a recombinant human serum albumin (rHSA) excipient increased stability outside of the cold chain dramatically [31]. While exact mechanisms underpinning this stability have not been investigated in this context, this is a widely used excipient in multiple vaccine formulations, and its strong antioxidant capacity due to free thiol moieties is believed to contribute [37].

Building on that prior work, here we present results from a Phase I clinical trial (ACTRN 12622000597796), with the primary objective being to evaluate the safety and tolerability of HexaPro delivered via the Vaxxas HD-MAP. As a secondary objective, we assessed the immunogenicity by measuring IgG and IgA responses, virus neutralisation, and antigen-specific cell-mediated immunity induced by HexaPro.

## 2. Materials and Methods

### 2.1. Study Design

This study was approved by the Bellberry Human Research Ethics Committee, acknowledged by the Therapeutic Goods Administration (TGA) via the clinical trials notification (CTN) pathway, and conducted in accordance with the applicable ICH Good Clinical Practice (GCP E6 R2) guidelines, the National Health and Medical Research Council (NHMRC) National Statement on Ethical Conduct in Research Involving Humans (2007), and the Declaration of Helsinki (1964) as most recently revised in Fortaleza (2013). The trial was registered with the Australian New Zealand Clinical Trials Registry (trial ID ACTRN 12622000597796). All laboratory investigators were blind to treatment and participant allocation. The primary objective was to measure the safety and tolerability of HexaPro delivered by HD-MAP in comparison to uncoated HD-MAP controls. Exploratory outcomes were to evaluate the immune responses to HD-MAP application via antibody titre measurements and virus neutralisation assays.

Healthy male and female (non-pregnant and non-nursing) subjects aged 18–50 years, with a body-mass index (BMI) of 18–32 kg/m^2^, were recruited into this single-centre, randomised, double-blinded, placebo-controlled study, conducted at the University of the Sunshine Coast Clinical Trials Center (Sippy Downs, QLD, Australia). This was a fourth-dose study, with all subjects having previously received three doses of a COVID-19 vaccine. The first two of these could have been any combination of Comirnaty^®^ (Pfizer, New York, NY, USA and BioNTech, Mainz, Germany), Spikevax (Moderna Inc., Cambridge, MA, USA), or Vaxzevria (AstraZeneca, Cambridge, UK), but the third dose was either Comirnaty or Spikevax and was administered at least four months prior to the commencement of the study. Each vaccine was monovalent, targeting only the original SARS-CoV-2 strain. This study design was chosen as it best reflected the vaccination status of the population at the time of the study and will assess the ability of HD-MAP vaccination to serve as a booster regime in a population with pre-existing immunity.

### 2.2. HD-MAP Manufacture and Vaccine Coating

The HD-MAPs used in this study were manufactured by injection moulding of a polymer to produce polymer patches with a micro-projection array area of 0.64 cm^2^. Each HD-MAP has approximately 1700 microprojections of ~350 µm in height and 120 µm in width at the base, stepping to a point of <20 µm. Clinical-grade HexaPro antigen was supplied by ExcellGene SA, Monthey, Switzerland, and stored at −80 °C prior to use. Subsequently, gamma-irradiated HD-MAPs were aseptically coated by dispensing vaccine to the tips of the microprojections using the “MJet”, a bespoke instrument and process patented by Vaxxas. Prior to HD-MAP coating, and to ensure antigen stability and potency during coating and subsequent storage, HexaPro was formulated with rHSA (Exbumin, which is free from any animal- or human-derived material, Invitria, Junction City, KS, USA, Cat#777HSA097) [31] and filtered through a 0.22 µm filter. Each HD-MAP was coated with 30 µg of formulated HexaPro to deliver an estimated 15 µg of antigen. The doses were determined using ex vivo porcine skin (described below) to be ~50% of the coated dose. After vaccine coating, the HD-MAPs were placed within an integrated applicator device containing a dome spring and molecular sieve desiccant (CSP Aptar, Cat#X-2626-610). Applicator devices were heat-sealed in a foil pouch that acted as the sterility and moisture barrier.

### 2.3. Delivered Dose Determination

To determine the vaccine dose delivered by HD-MAP into the skin, a radioactive tracer Albumin [methyl-14C] methylated (bovine serum) (5 µCi/mL) in 0.01 M NaH_2_PO_4_ (American Radio-labelled Chemicals, St Louis, MO, USA, Cat#ARC-0422-5 Ci) was co-formulated into the vaccine solution. HD-MAPs were coated with ^14^C-BSA and varying vaccine concentrations. After 2 min application time of HD-MAPs onto ex vivo Large White pig skin, MAPs were removed from the skin and placed into scintillation vials (Merck, Darmstadt, Germany; Cat# V6880) prefilled with PBS (Invitrogen, Waltham, MA, USA, Cat#003002). The skin’s surface was swabbed with a PBS-dampened cotton swab and placed into separate PBS-containing scintillation vials. Post HD-MAP application, skin samples were excised and collected into scintillation vials with 1 mL of Solvable™ (Revvity Inc., Waltham, MA, USA, Cat#6NE9100) to solubilise tissue. Control MAPs (unapplied, vaccine-coated) were placed into PBS-containing scintillation vials. MAP and swab samples were eluted overnight at room temperature on an orbital shaker (120 rpm), whilst tissue solubilisation occurred at 60 °C stationary overnight. The next day, 10 mL of Ultima Gold™ Scintillation liquid (Revvity Inc., Waltham, MA, USA, Cat#6013329) was added to each vial and vigorously vortexed. Calibration of beta-emitter including ^14^C standards was run immediately prior to the samples on the scintillation analyser (Tri-Carb 4910TR Liquid Scintillation Counter, Revvity Inc., Waltham, MA, USA), where beta emission was measured for 10 min per sample and analysed using the Quanta Smart software V4.00. Scintillation counts as disintegration per minute (DPM) were converted into percentages with readings of elution controls set to 100%. Delivery efficiency percentage was calculated as = [[average control MAPs (n = 3) − (swab + remaining ^14^C from applied HD-MAP)]/average of control MAPs] × 100.

### 2.4. Scanning Electron Microscopy

Formulation coated onto MAP projections was visualised via scanning electron microscopy (SEM) utilising a Hitachi TM3030 plus or a TM4000 plus machine (Hitachi High-Technologies Corporation, Tokyo, Japan). The SEM images form part of an in-process qualitative targeting check during HexaPro HD-MAP manufacture and batch release. MAPs were also assessed post-application to observe the residual formulation remaining after application to skin (for informational purposes only). The MAPs remained subject- and cohort-blinded during this process and were subsequently grouped per subject before immunogenicity results were received.

### 2.5. Thermostability Analysis of HexaPro on HD-MAPs

HexaPro-coated HD-MAPs were stored at 5 ± 3 °C, 25 ± 2 °C/60 ± 5% relative humidity (RH), and 40 ± 2 °C/75 ± 5% RH (as per ICH Q1A(R2) guidelines) for 12 months. To prepare for potency assay of the HexaPro, the coated vaccine formulation on the HD-MAP surface was eluted into buffer. For the elution, MAPs were first equilibrated at room temperature for at least 30 min. To elute, the spigot was removed from the MAPs, they were placed in 24-well plates, and DPBS was added onto the projection side of the MAP surface until the MAP was hydrated. Each MAP was rinsed 50 times via pipette dispensing. Eluates were then analysed for HexaPro integrity and antigenicity by capture ELISA using recombinant mAbs, which were produced in-house [31]. DH1047 was used as a capture antibody and was coated in DPBS at 1 µg/mL onto Nunclon ELISA plates overnight at 4 °C and washed with 1 x PBST buffer (137 mM NaCl, 10 nM Phosphate, 0.05% Tween-20, pH 7.2). Plates were then blocked for a minimum of 2 h shaking with 1% skim milk powder PBST blocker. Plates could also be prepared in advance and frozen at −20 °C during the blocking stage and used within 6 months of preparation. All washes were performed with 1 × PBST buffer. HexaPro standards, MAP vaccine coating solution, and MAP eluates were all prepared to ~100 µg/mL and incubated overnight at 37 °C. Standards were performed in duplicate from 8 µg/mL to 7.8 ng/mL. Samples and controls were performed in duplicates from 2 µg/mL to 62.5 ng/mL. All dilutions were performed using skim milk blocking buffer as diluent, and sample incubation was for 60 min at room temperature. After another wash step, the detection antibody (HRP-conjugated S309) was added. The HRP conjugation was performed using a Lightning-link kit as per the manufacturer’s instructions (Abcam, Cambridge, UK, Cat#Ab102890). BioFX 3,3′,5,5′-tetramethylbenzidine (TMB, SeraCare Life Sciences, Milford, MA, USA, Cat#5120-0075) substrate was used for detection and followed by an acid stop, with the plate read at an absorbance of 450 nm. The HexaPro DS standard curve was constructed to interpolate the unknowns.

HexaPro identity was further confirmed via size exclusion chromatography on an Agilent Prime II high-pressure liquid chromatography (HPLC) system. Samples were loaded onto an AdvanceBio SEC 300A 2.7 µm 7.8 × 150 mm column equipped with an AdvanceBio SEC 300A 2.7 µm 7.8 × 50 mm guard column (Agilent Technologies, Santa Clara, CA, USA, Cat#PL1180-1302). PBS (100 mM sodium phosphate, 150 mM sodium chloride, pH 6.5) was used as the mobile phase. AdvanceBio SEC 300A protein standards and BSA (2 mg/mL) were used to normalise data (Agilent, Technologies, Santa Clara, CA, USA, Cat#5190-9417). All samples were run at a flow rate of 1.25 mL/min. The absorbance of the samples was monitored at 280 nm.

The bicinchoninic acid (BCA) assay was used to quantify the total protein concentration of MAP elution samples containing both HexaPro and rHSA. Briefly, this assay was performed using the Pierce™ BCA protein kit (ThermoFisher Scientific, Waltham, MA, USA, Cat#A55864). In a 96-well plate, 50 µL of samples (including standards and controls) were run in triplicate, with the addition of 200 µL working reagent per well. The microplate was incubated on the bench at room temperature for 1 h, and the plate was subsequently read at 562 nm. Blank corrected data was used to generate a standard curve of known concentrations to interpolate the total protein concentration of samples in Prism (GraphPad Software, San Diego, CA, USA).

### 2.6. Vaccination Procedure

Each HD-MAP application was performed on the subject’s upper arm overlying the deltoid muscle. The potential application site on the upper arm was first examined to ensure it was free from tattoos, sunburn, scarring, skin conditions, and heavy hair. Next, the HD-MAP and integrated applicator device were removed from the sealed foil pouch and the foil seal on the skin-facing side of the applicator removed also. The device was applied to the upper arm and activated to apply the HD-MAP to the skin. The device was held in place for 2 min +/− 10 s before being removed. All vaccination sites were assessed for local reactogenicity at 1h and 2 h after HD-MAP application and again at each clinical review (days 3, 7, 28, 56, and 90) and photographs were collected (Apple iPhone 6). The local reactions were examined for erythema, treatment site visibility scored not visible or on a scale of 1 to 4 (1: site barely perceptible, 2: site is visible, 3: moderate to severe redness and/or discolouration, and 4: severe, beet-redness to slight eschar formation), redness/discolouration extent (whether it is circumscribed to the application area), swelling and whether it is oedema or induration, swelling extent (whether it is circumscribed to the application area), and skin flaking.

### 2.7. Spike-Binding Antibody Assays

Blood and saliva samples were collected on days 0 (pre-vaccination), 7, 28, 56, and 90. Serum was isolated from the blood and stored at −80 °C until analysis. Saliva was collected using a saliva collection kit Salivette (Sarstedt, Nümbrecth, Germany, Cat# 51.1534). Anti-SARS-CoV-2 Spike protein IgG or IgA antibody levels were quantified using a multiplex serology MSD assay (Meso Scale Discovery, Rockville, MD, USA) performed at Cerba (Rotterdam, The Netherlands). Samples were tested against the V-PLEX SARS-CoV-2 IgG or IgA assay panel 2 (containing wildtype spike protein, RBD or N protein, Meso Scale Discovery Cat#K15383U and Cat#K15385, respectively) or V-PLEX SARS-CoV-2 IgG panel 34 (against BA.2.75, XBB.1 and XBB.1.5, Meso Scale Discovery Cat#15690U). Serum and saliva samples were added in duplicate in 96-well plates coated with specific antigens, respectively. After binding of specific IgG antibodies in the serum or saliva samples to the antigens, detection was performed using an anti-human MSD SULFO-TAG antibody conjugate. The emitted light was measured using a Meso SECTOR S 600MM device (Meso Scale Discovery). IgG antibody levels against SARS-CoV-2 Spike protein for variants BA.2.75, XBB.1, and XBB.1.5 were reported as assay units (AU/mL). IgA antibody levels against wildtype SARS-CoV-2 Spike, RBD, and NP were reported as assay units (AU/mL).

### 2.8. Virus Neutralisation Assays

Serum anti-SARS-CoV-2 neutralising antibody levels were determined using a validated proprietary assay (Cerba, Rotterdam, The Netherlands). Briefly, in this assay, a serial dilution in triplicate of a serum sample in infection medium was mixed with a fixed input amount of infectious SARS-CoV-2 virus and incubated for 1 h. The input virus used was either isolate Germany/BavPat1/2020 as a wildtype D614G variant, isolate hCoV-19/Netherlands/NH-RIVM-72291/2021 as variant Omicron BA.1, or isolate hCoV-19/Netherlands/VCB-20220303-1/2022 as variant Omicron BA.2.

The mixture was then added to subconfluent monolayers of VeroE6 cells (ATCC CRL-1586) and incubated for 1 h, after which the inoculum was removed and replaced by infection medium containing carboxymethyl cellulose (CMC). Cells were then incubated for 20–24 h, subsequently fixed with a formalin solution, and the presence of viral microplaques was detected by immunostaining using an anti-nucleoprotein antibody, a peroxidase conjugate, and TrueBlue staining. Microplaques were imaged and counted using an SX Ultimate-V Analyzer (Cellular Technologies Limited, Shaker Heights, OH, USA). The neutralisation titres were calculated from these data following the method of Zielinska et al. [38].

### 2.9. cT_FH_ and T Cell Activation Induced Marker Assay

Cryopreserved PBMCs were thawed at 37 °C, washed twice in RF10 (RPMI-1640 with 10% foetal calf serum, penicillin, and streptomycin), and rested for 2 h. Cells were seeded in a 96-well plate at 2 × 10^6^ cells/well and stimulated with 1 µg/mL of the PepTivator SARS-CoV-2 Prot_S Complete wildtype spike peptide pool (Miltenyi Biotec, Bergisch Gladbach, Germany, Cat #130-126-701) or vehicle control (water). Anti-CD154 APC-Cy7 (BD, TRAP1; 1:300) antibody was added to all wells. Cells were incubated for 20 h at 37 °C in the dark, before being washed, stained with live/dead aqua viability dye as per manufacturer’s instructions (ThermoFisher Scientific; Cat#L34957), and stained with the following antibody surface staining cocktail for 30 min at 4 °C: BV510 Mouse Anti-Human CD20 (BD; 27H; 1:100); BUV805 Mouse Anti-Human CD3 (BD; SK7; 1:100); Brilliant Violet 605™ anti-human CD4 Antibody (Biolegend, San Diego, CA, USA, RPA-T4; 1:100); Brilliant Violet 650™ anti-human CD8a Antibody (Biolegend; RPA-T8; 1:400); FITC anti-human CD45RA (Biolegend; HI100; 1:300); Alexa Fluor^®^ 647 anti-human CD197 (CCR7) Antibody (Biolegend; G043H7; 1:20); CD185 (CXCR5) Monoclonal Antibody (eBioscience, San Diego, CA, USA; MU5UBEE; 1:50); Brilliant Violet 421™ anti-human CD137 (4-1BB) Antibody (Biolegend; 4B4-1; 1:100); PerCP/Cyanine5.5 anti-mouse CD134 (OX-40) Antibody (Biolegend; BER-ACT35; 1:50); APC-Cy™7 Mouse Anti-Human CD154 (BD Biosciences, San Jose, CA, USA; TRAP-1; 1:300); and PE/Dazzle™ 594 anti-human CD69 Antibody (Biolegend; FN50; 1:200). Cells were then washed, fixed in 1% Cytofix fixation buffer (BD Biosciences), and acquired on an LSR Fortessa flow cytometer (BD Biosciences). cT_FH_ were selected to be CD3^+^ (to exclude B cells), CD4^+^ (to exclude CD8^+^ cells), and CXCR5^+^ (to identify cT_FH_ cells).

### 2.10. Quantitation and Statistical Analysis

The primary objective was to measure the safety and tolerability of HexaPro administration by HD-MAP. Demographic and clinical measurements were to be summarised by frequencies for categorical variables and by mean, standard deviation, and range for continuous variables. The significance level was assumed to be *p* < 0.05 (two-tailed). Results from the parametric analysis are presented in the main text, tables, and figures. As this was an early phase study, no adjustments were made for multiplicity for any of the analyses, including immunogenicity assays. Safety treatment site assessments and immune responses before and after a single treatment were to be compared within and between groups. Analyses were performed and prepared in GraphPad Prism version 10.1.1.

## 3. Results

### 3.1. Trial Profile and Study Procedures

**Figure 1:**
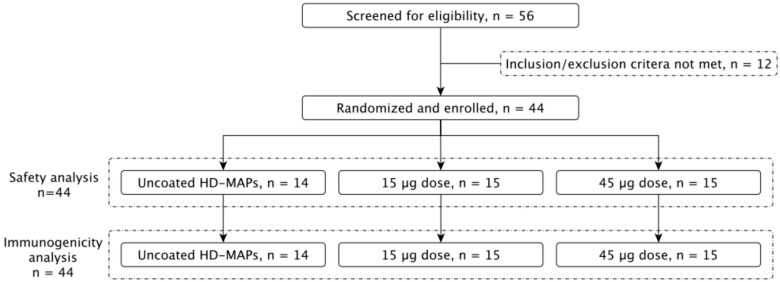
Clinical trial profile. A total of 56 subjects were screened for eligibility, of which 44 were enrolled. Subjects were split into 3 groups, receiving 3 HD-MAPs each, resulting in either a 0, 15, or 45 µg dose of vaccine.

Between 20 October 2022 and 27 February 2023, 44 healthy subjects between the ages of 18 and 50 years were enrolled into the study and vaccinated as per the grouping in Figure 1 and Table 1. The trial consisted of three groups, each subject receiving a total of three HD-MAPs, either active (i.e., coated with vaccine) or inactive (i.e., application of uncoated HD-MAPs). This was a “fourth COVID vaccine dose” study, where subjects had received three prior COVID-19 vaccines, where at least the previous two vaccinations were mRNA vaccines (Comirnaty, Pfizer or Spikevax, Moderna) no less than four months prior to enrolment. Group 1 (uncoated HD-MAP group, n = 14) received three uncoated HD-MAPs, group 2 (low-dose treatment group, n = 15) received one active and two inactive HD-MAPs, for an estimated delivered dose of 15 µg of HexaPro, and group 3 (high-dose treatment group, n = 15) received three active HD-MAPs, delivering an estimated 45 µg of HexaPro. This approach was chosen such that subjects received an equal number of HD-MAP applications. Subjects had serum and saliva collected on days 0, 7, 28, 56, and 90 post-vaccination to monitor for SARS-CoV-2-specific IgG and IgA, respectively. PBMC were collected on day 0 and day 7 for analysis of T cell responses.

### 3.2. Summary of Adverse Events

The vaccination was well tolerated in all groups, and no safety issues were identified. Throughout the trial period, all adverse events (AE) were deemed treatment-emergent AEs (TEAEs, Table 2), where 36 (81.8%) subjects experienced a total of 98 TEAEs. Within the TEAEs, a total of 25 subjects recorded 50 TEAEs (56.8%) that were considered to be related to the study treatment (HD-MAP) (Table 3). Of those, 17 subjects experienced localised reactions (38.6%) and 14 subjects experienced systemic reactions (31.8%). Most adverse events were mild or moderate in severity. There was only one severe TEAE (lower abdominal pain) occurring in one (2.3%) subject in Group 3 (45 µg), which was deemed unlikely to be related to the study medication. There were no life-threatening or fatal adverse events or adverse events resulting in withdrawal of the study treatment. A summary of adverse events can be found in Table 2 and Table 3.

### 3.3. HD-MAP Coating, Delivery Efficiency, and Thermostability

**Figure 2:**
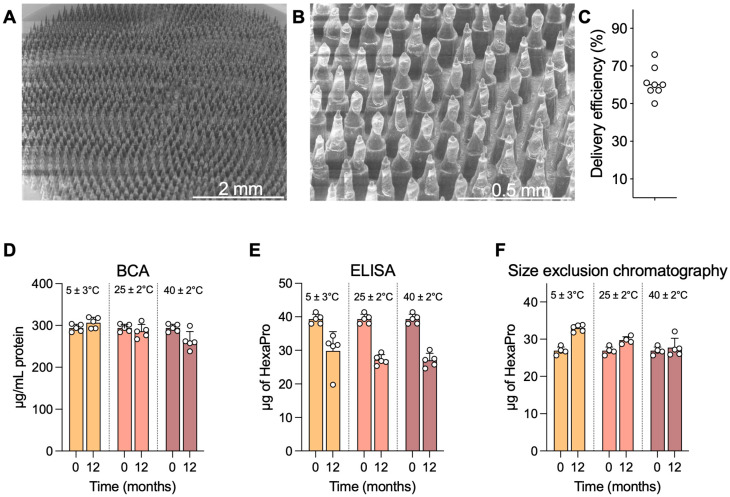
HD-MAP coating and thermostability. (**A**) Scanning electron microscopy image of an HD-MAP coated with the HexaPro vaccine formulation at 30× or (**B**) 150× magnification. HD-MAPs were coated with vaccine and stored for 12 months at the temperatures indicated. (**C**) Delivery efficiency into ex vivo pig skin as assessed by ELISA. Integrity of HexaPro was assayed via (**D**) bicinchoninic acid assay for total protein, (**E**) capture ELISA using spike-specific mAbs DH1047 and S309, and (**F**) size-exclusion chromatography for trimeric spike protein. Data are presented as mean of 5 replicate HD-MAPs. Error bars represent the standard deviation.

The vaccine coating morphology on the microprojections of the HD-MAP is an important consideration, as it can impact potential delivery of the vaccine payload into the skin. During development for the clinical batches, preclinical work assessed delivery of the vaccine payload into membrane (in vitro) and into ex vivo pig skin. HD-MAPs were coated with excipient formulated HexaPro and qualitatively examined via scanning electron microscopy (SEM) (Figure 2A,B). The HexaPro formulation coated well onto the HD-MAPs and, post-delivery to the skin of trial subjects, removal of the majority of the vaccine coating was confirmed by SEM with an estimated skin delivery efficiency of 50–76% measured by ELISA (Figure 2C), confirming the preclinical data from ex vivo pig skin. Moving forward, 50% delivery efficiency was used as the lowest expectation of delivery efficiency. Some material did remain on the base of the microprojections as expected from the preclinical work.

HexaPro HD-MAPs were stable at elevated temperatures. HexaPro was coated onto HD-MAPs in an rHSA-containing formulation, which had previously afforded stability at elevated temperatures over a 1-month period [31]. To expand on this, HD-MAPs were coated and stored protected from moisture at 5 ± 3 °C, 25 ± 2 °C/60 ± 5% relative humidity (RH), and 40 ± 2 °C/75 ± 5% RH (as per ICH Q1A(R2) guidelines) for 12 months and assayed for HexaPro integrity via bicinchoninic acid (BCA) assay, ELISA potency, and size-exclusion chromatography. The BCA assay measures total protein, including the excipient rHSA, to confirm that all the material is being eluted and that an apparent drop in stability is not due to material being retained on the HD-MAP. In this BCA assay, minimal differences in protein recovery were seen at any temperature, with only a slight drop seen at the 40 ± 2 °C samples, likely indicating issues in recovering dried material off the HD-MAP surface into solution (Figure 2D). In the ELISA, which utilised spike-specific mAbs S309 and DH1047 (both broadly reactive spike-specific mAbs with high neutralising potency) in a sandwich capture format, a drop in recovered HexaPro quantity was observed in all temperatures, though this did not differ between 5 °C and the elevated temperatures (Figure 2E). When HexaPro was analysed via size-exclusion chromatography to assess for trimeric HexaPro, indicative of correct protein folding or aggregation or degradation occurring during storage, data showed trimeric HexaPro was recovered at all temperatures (Figure 2F).

### 3.4. HD-MAP Application Site Reactions and Resolution

**Figure 3:**
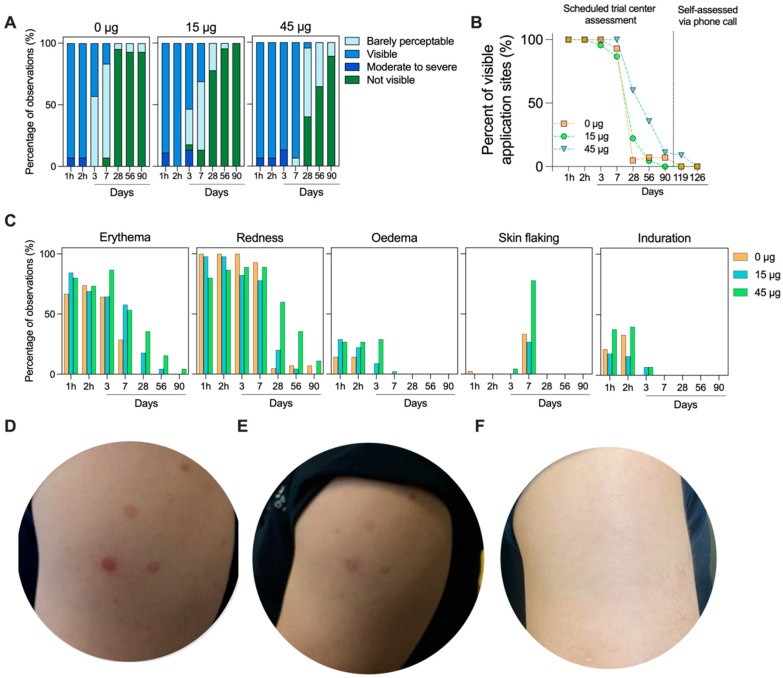
HD-MAP application site resolution over time. (**A**) Visibility scores for each application site (n = 3 sites/subject, n = 14 subjects for 0 µg group, n = 15 subjects for 15 and 45 µg groups) throughout the study. (**B**) Percentage of visible application sites and (**C**) skin reactogenicity scoring observed for each group throughout the trial period. Representative skin images from (**D**) day 3, (**E**) day 7, and (**F**) day 28. All subjects received three HD-MAP applications. In this instance, the subject received one active HD-MAP (lower left) and two uncoated HD-MAPs (upper and lower right).

All application sites were visible on the subject’s skin within 1 h of the HD-MAP being removed, providing clear evidence of HD-MAP engagement with the skin. By day 3 in the uncoated HD-MAP group, over half (57.1%) of the application sites were deemed barely visually perceptible (Figure 3A). Whereas, in the high-dose treatment group, all application sites remained visible at day 3. This trend continued at day 7, with most active HD-MAP sites remaining visible, though 76.2% of the uncoated group’s application sites were deemed barely perceptible. By day 28, the 15 µg group showed complete resolution in 77.8% of application sites, compared to 40% in the high-dose treatment group and 95.2% in the uncoated HD-MAP group (Figure 3A,B). By days 56 and 90, all application sites were deemed not visible or barely perceptible. In follow-up phone calls with the subjects, self-reported assessment revealed all application sites as not visible by day 126 in all groups (Figure 3B).

The area of skin receiving the HD-MAP was examined for local reactions, including erythema (defined by skin blanching), treatment site visibility (scored on a scale of 1 to 4, from barely visible to beet-redness), the redness/discoloration extent (whether it is circumscribed to the application site), swelling (oedema or induration and extent), and skin flaking. Erythema (defined by skin blanching) was seen in all groups within 1 h. This was coupled with application site visibility and oedema (Figure 3C). Reactions in general were similar between the three groups, though the erythema, visibility, and oedema took longer to resolve in the 45 µg group due to the greater number of active HD-MAP applications in this group. At day 7, skin flaking was observed in all groups, though it occurred at a higher rate in the 45 µg group. Representative skin images (Figure 3D,F) illustrate the slightly higher visibility in the active MAP groups compared to uncoated HD-MAP sites, though, by day 90, all sites were completely resolved.

### 3.5. HexaPro Delivered by the HD-MAP Induces Spike-Binding IgG

**Figure 4:**
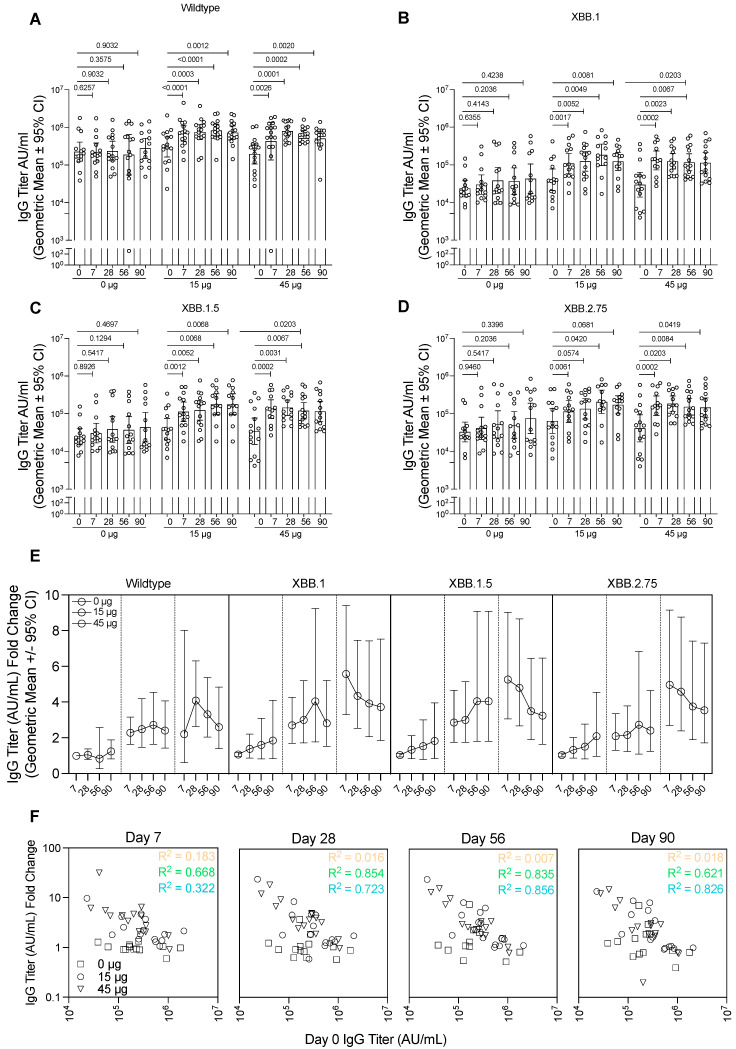
Immunogenicity analysis of serum samples against wildtype and variant spike proteins. Serum collected from subjects from each group were analysed for IgG against (**A**) wildtype spike protein, matching the vaccine antigen, (**B**) XBB.1 spike protein, (**C**) XBB.1.5 spike protein, or (**D**) XBB.2.75 spike protein. Data presented as the geometric mean with error bars showing the 95% confidence intervals. *p*-values indicated from the Wilcoxon matched-pairs signed rank test. (**E**) IgG titre for each of the spike proteins represented as a fold-change relative to day 0 titres for each of the groups. Data presented as the geometric mean with error bars showing the 95% confidence intervals. (**F**) The relationship between the spike-specific IgG titre prior to vaccination (day 0) and the titres at each of the timepoints after vaccination. Dotted lines represent least squares regression for each group, with R squared values indicated. AU, antibody units.

Induction of spike-binding antibodies by HexaPro HD-MAP vaccination was assessed using the Mesoscale Discovery V-PLEX assays against a panel of SARS-CoV-2 spike proteins (Figure 4). These proteins were selected to either match the vaccination strain (termed wildtype, as it matches the spike protein from early isolates of SARS-CoV-2) or represent recently circulating variants (XBB.1, XBB.1.5, and XBB.2.75). Nucleocapsid was included in the panel to detect antibodies induced by SARS-CoV-2 infection rather than vaccination. Subjects were screened for confirmed SARS-CoV-2 infections prior to the trial period but relied on rapid antigen test (RAT) testing at each site visit to detect infection during the trial. Increases in nucleocapsid-specific serum IgG titres were seen for one subject in the 15 µg group, one subject in the 45 µg group, and three subjects in the uncoated HD-MAP group (Figure S1). These subjects were not excluded from the immunogenicity analysis. No changes in nucleocapsid-specific IgA titres were seen in saliva samples (Figure S2).

In the group receiving uncoated HD-MAPs only, no significant changes in IgG concentration against the wildtype spike protein were seen in any timepoint relative to day 0 (Figure 4A). In the 15 µg group, the IgG concentration was significantly higher relative to day 0 by day 7 (*p* < 0.0001). From day 7 until the final timepoint at day 90, the IgG concentration remained elevated, though maintained levels that were statistically significantly higher relative to day 0 (*p* = 0.0003, *p* < 0.0001, and *p* = 0.0023 relative to day 0 for days 28, 58, and 90, respectively). In the highest dose group (45 µg), the trend was similar, with significant increases in IgG titre at day 7 (*p* = 0.0026 when compared to day 0), which peaked slightly at day 28 (*p* = 0.0001 relative to day 0) and remained constant on days 56 (*p* = 0.0002) and 90 (*p* = 0.0020) relative to day 0.

SARS-CoV-2 is continually evolving, with new variants emerging regularly; therefore, it is important to induce a broad immune response against diverse virus strains. Thus, we tested the IgG titre as before against the spike protein from recently circulating strains XBB.1, XBB.1.5, and XBB.2.75. While IgG titres against these proteins were overall lower than those seen when testing with the cognate vaccine antigen strain, the HD-MAP vaccination regime showed similar trends of boosting as it did for the wildtype IgG titres (Figure 4B–D). In both the 15 and 45 µg groups, against all variants tested, there was a statistically significant increase by day 7 compared to day 0 titres, which remained at a similar level until the endpoint on day 90. The control group showed no significant changes in IgG titre relative to day 0 against these proteins. There was, however, a trend for increasing antibody titres against variants XBB.1, XBB.1.5, and XBB.2.75 but not wildtype in the control group, due to four subjects who became infected during the trial (as indicated by anti-nucleocapsid antibodies).

As this was a fourth-dose study, subjects had prior exposure to the spike protein through infection or vaccination, though all subjects in all groups had similar levels at day 0 (Figure S3). As such, it was of interest to examine the HD-MAP’s ability to boost the immune response. To do this, we calculated the fold-change in IgG titre relative to day 0 titre (Figure 4E, Supplementary Table S1). This revealed minimal changes in the IgG titre over the study period for the 0 µg group. However, in those receiving active MAPs, there was clear boosting of the IgG titres for all spike proteins tested. There was also a clear correlation with higher IgG fold-change occurring in subjects with lower incoming day 0 IgG titres, especially apparent on days 28 and 56 (Figure 4F, R^2^ values of 0.854 and 0.723 for 15 and 45 µg groups on day 28 and 0.835 and 0.856 for those same groups on day 56). This was also tested against the XBB.1, XBB.1.5, and XBB.2.75 spike proteins, where similar trends were seen (Figure S4).

Delivery of vaccines via the HD-MAP has been shown to induce mucosal immune responses in preclinical animal models [31]. Therefore, saliva was collected from the subjects at the same times as serum and analysed for anti-spike and anti-RBD IgA levels (Figure S4). There were no significant differences seen in any of the groups relative to their day 0 titres.

### 3.6. HexaPro HD-MAP Vaccination Induces Neutralising Antibody Responses Against Recently Circulating Variants

**Figure 5:**
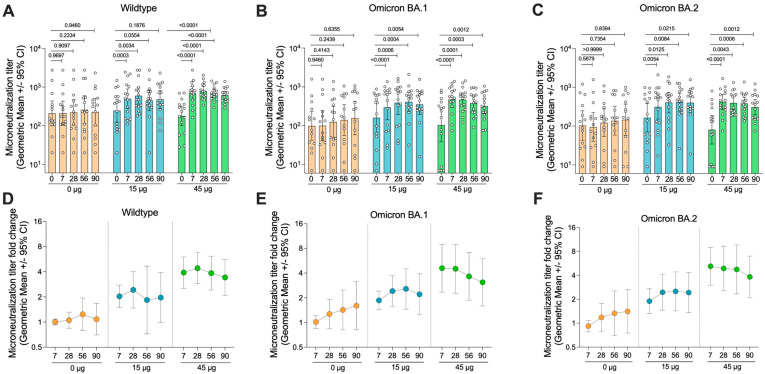
Neutralisation activity of serum from vaccinated subjects. (**A**) Serum collected from subjects from each group was analysed for their neutralisation activity against wildtype SARS-CoV-2 (Germany/BavPat1/2020), (**B**) Omicron BA.1 SARS-CoV-2 (hCoV-19/Netherlands/NH-RIVM-72291/2021), and (**C**) Omicron BA.2 SARS-CoV-2 (hCoV-19/Netherlands/VCB-20220303-1/2022). (**D**–**F**) Fold-change in microneutralisation titre relative to day 0 pre-vaccination titre for each strain. Data presented as the geometric mean with error bars showing the 95% confidence intervals. *p*-values indicated from the Wilcoxon matched-pairs signed rank test.

To assess the functionality of the induced antibody responses, neutralising antibody titres in the serum was tested against an early isolate of SARS-CoV-2, matching the antigen used for vaccination, as well as two recently circulating Omicron variants, BA.1 and BA.2 (Figure 5). In general, the neutralisation data mirrored the MSD V-PLEX data, with the control group showing no significant increase in neutralisation titre on any day relative to day 0 (*p* > 0.05 for all). In the group receiving 15 µg of HexaPro, significant increases in neutralisation titres were seen again by day 7 against all variants tested. Titres remained elevated until day 90 for all strains, although, for the wildtype virus, there was a drop below statistical significance on day 90 relative to day 0 (*p* = 0.1876). The 45 µg group again showed similar trends, with slightly higher neutralisation titres relative to the 15 µg group. Furthermore, in the 45 µg group, statistical significance was maintained (*p* < 0.001) for all time points relative to day 0. Indeed, the average fold-change in neutralisation titre appeared slightly higher in the 45 µg dose compared to the 15 µg dose (Figure 5D).

There was an increase in the titre of neutralising antibodies against the Omicron BA.1 and BA.1 strains in the placebo group. The increase was not significant and was attributed to four subjects in this group who had asymptomatic SARS-CoV-2 infection after day 0 of the trial, as indicated by seroconversion to N protein (Figure S1). This explanation is supported by the observation that the increase in neutralising titres was larger against the recently circulating variants (BA.1 and BA.2) compared with the wildtype strain.

### 3.7. HexaPro HD-MAP Vaccination Did Not Induce a Measurable Increase in Antigen-Specific T Cells

**Figure 6:**
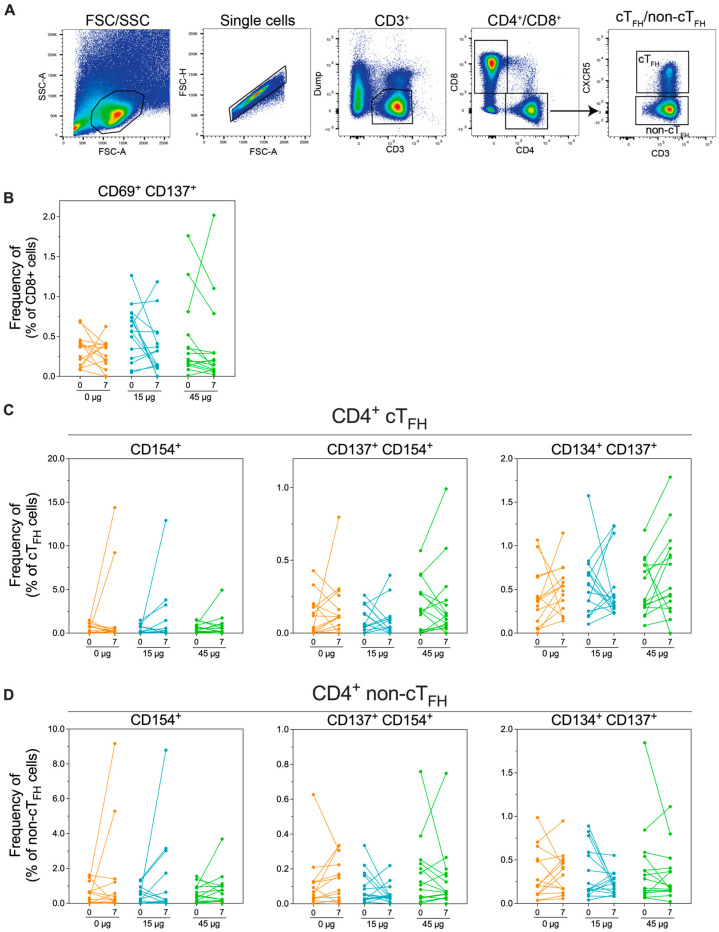
Analysis of SARS-CoV-2 spike-specific cellular immunity in vaccinated subjects. Peripheral blood mononuclear cells (PBMCs) were collected from subjects on day 0 (pre-immunisation) or day 7 post-immunisation. PBMCs were stimulated with overlapping peptides spanning the spike protein sequence before staining for activation markers. (**A**). Gating strategy used for PBMCs. (**B**) Frequency of CD8^+^ cells expressing the activation markers CD69 and CD137. (**C**) Frequency of CD4^+^ circulating T follicular helping (cT_FH_) cells, defined as CD4^+^ CXCR5^+^ or (**D**) CD4^+^ non-cT_FH_ cells (defined as CD4^+^ CXCR5-) expressing the activation markers CD154, CD137, and/or CD134.

T cell responses have been proven to be important for controlling and preventing virus infections, including SARS-CoV-2. Therefore, to assess the induction of T cell responses to HexaPro HD-MAP vaccination, we utilised the activation-induced marker (AIM) assay [39]. Peripheral blood mononuclear cells (PBMCs) from subjects were taken on days 0 (pre-vaccination) and day 7 and stimulated with a peptide pool spanning the SARS-CoV-2 spike protein. PBMCs were then stained for expression of lineage-specific markers (CD4 and CD8) and several AIMs (CD137, CD134, and CD154 for CD4^+^ cells and CD69 and CD137 for CD8^+^ cells). In addition, the markers were chosen to define circulating T follicular helper (cT_FH_) cells (CD4^+^ CXCR5^+^), believed to be important for the establishment of long-term immune memory (Figure 6A). There were no clear trends in the upregulation of any of the AIMs assayed in either CD8^+^ (Figure 6B), CD4^+^ cT_FH_ (Figure 6C), or CD4^+^ non-cT_FH_ cells (Figure 6D). Some individuals had unexpectedly high cell frequencies at day 0 or day 7; however, these occurred indiscriminately in all groups.

## 4. Discussion

Vaccine equity and global distribution remain a problem, highlighted by the COVID-19 pandemic. MAP technology has the potential to overcome many of these barriers to equitable vaccine access, particularly for low- and middle-income countries (LMICs). In this Phase I trial, we evaluated a novel SARS-CoV-2 spike protein vaccine candidate, HexaPro, delivered to healthy subjects using the HD-MAP.

The HexaPro HD-MAP vaccine was safe and well-tolerated by all subjects, meeting the study’s primary objective. Skin reactions were more frequent in the 45 µg vaccine dose, likely due to the higher number of active HD-MAPs applied per subject, though application of the HD-MAP itself did cause skin reactions, indicative of microneedle penetration of the skin. A greater proportion of skin reactions persisted through days 28 and 56 in the 45 µg group compared to the 15 µg or uncoated HD-MAP groups. Future studies will use a single HD-MAP per dose to assess local reactogenicity in relation to dose. Redness, erythema, and swelling also took longer to resolve in the active groups, with more skin flaking observed at day 7. This could be indicative of localised immune responses to the vaccine antigen. Indeed, prior research with the HD-MAP coated with an inactivated influenza virus vaccine showed that more cellular infiltrates, including T cells, were seen with an active, vaccine-coated HD-MAP compared to an uncoated HD-MAP [40]. It was hypothesised that the existing immunity of the subjects influenced this infiltration. Therefore, it is possible that prior immunity to the spike protein in the subjects resulted in rapid recruitment of antigen-specific immune cells, leading to a prolonged (albeit transient) skin reaction. It is of course possible that this response is due to the delivery of an immunogenic antigen, rather than pre-existing immunity. This could be examined by delivery of a vaccine to subjects that are completely naïve to the antigen and examining the skin responses. Examination in subjects with varying skin pigmentation and age is also of interest to understand the skin response and ensure vaccine efficacy is maintained. Nonetheless, it is important to consider the impact of visible vaccine delivery sites on uptake of this vaccine technology by consumers and healthcare professionals, with future HD-MAP designs carefully balancing immunological efficacy with vaccine site resolution time.

HexaPro HD-MAPs showed promising stability at 12 months, including at elevated temperatures (25 °C and 40 °C). The size exclusion chromatography analysis showed no change in trimeric HexaPro content, though the ELISA data showed a slight drop in HexaPro quantity at the 12-month mark. This could indicate the epitopes bound by the antibodies used in the capture ELISA format were altered by storage; however, this did not alter the oligomeric status of the protein in the size exclusion analysis. This stability improvement holds significant potential for enhancing vaccine access in LMICs and is a notable advancement over current COVID-19 vaccines, though vaccine efficacy assessment of HD-MAPs after these storage conditions is required to confirm stability. For comparison, Nuvaxovid (Novavax), another protein-based SARS-CoV-2 vaccine, has a reported 9-month shelf life when stored at 2–8 °C [41]. mRNA vaccines, on the other hand, are known to have stability issues, often requiring ultra-low temperature storage, though some efforts are underway to create lyophilised mRNA vaccines that can be stored at higher temperatures. While this observed stability is likely a combination of inherent stability of the HexaPro antigen combined with formulation and dry-coating on the HD-MAP itself, it is worth noting that similar stability profiles have been seen for other diverse vaccine types on the HD-MAP, including virus-like particles and live-attenuated measles-rubella vaccine [9]. This suggests some inherent stability is afforded by the formulation and HD-MAP coating process. Overall, the HD-MAP HexaPro vaccine offers significant stability improvements over the currently available vaccines.

As a secondary objective to this study, analysis of the IgG responses to various SARS-CoV-2 spike protein variants showed that the HD-MAP vaccination boosted responses. No substantial difference in boosting was seen between the 15 and 45 µg doses, suggesting that even lower doses may achieve similar immune responses. The boost in IgG response was also seen against highly divergent spike proteins from XBB.1, XBB.1.5 and XBB.2.75. This extended to functional neutralisation assays, where neutralisation was seen against Omicron BA.1 and BA.2 variants. This data is in line with preclinical mouse studies involving HexaPro HD-MAP delivery from our group, where HD-MAP delivery resulted in significant cross-neutralisation against highly divergent viral variants, which was greater in magnitude relative to intradermal injection [31]. There were several patients who were positive for nucleocapsid-specific antibodies, indicating prior infection (Supplementary Figure S1). This could impact the antibody titres measured in this study. Though, it is worth noting that the fold-change analysis (Figure 4E and Figure 5D–F) shows the control group has a slight increase in antibody titres against the variants in follow-up visits. However, it still demonstrates higher effects when antigen was included as compared to the 0 µg group. As the primary objective of this Phase I study was to assess the safety of the HD-MAP-delivered HexaPro vaccine, no needle-and-syringe comparator was included. It is not possible, therefore, to draw conclusions about the relative immunogenicity of HD-MAP-delivered HexaPro compared with approved SARS-CoV-2 vaccines such as Spikevax (Moderna Inc.), Comirnaty (Pfizer-BioNTech), or Nuvaxovid (Novavax Inc., Gaithersburg, MD, USA). Furthermore, although neutralising antibodies are regarded as a correlate of protection against SARS-CoV-2, threshold titres for protection against infection by wildtype or variants of concern have not been defined, again making comparisons with other vaccines difficult. Phase II studies and beyond should include an intramuscular injection of either the dose-matched HexaPro vaccine or another licensed vaccine. Such studies could also investigate optimisation of the coating and delivery to obtain efficiencies above the 50% observed in this study, including assessing whether the dose delivered into the skin is impacted by storage outside of the cold chain. This would lend more credence to the dose-sparing advantages of the HD-MAP. This would allow investigation into the previous observations of dose-sparing and immunogenicity improvements afforded by the HD-MAP, for example, like what was seen in a clinical trial of an influenza virus vaccine candidate [4].

We did not detect significant increases in the frequency of activated spike-protein-specific T cells or cT_FH_ cells following HD-MAP delivery of HexaPro. It could be that unadjuvanted HD-MAP-delivered HexaPro is not able to stimulate T cell responses. In previous mouse studies, HexaPro delivery with the saponin-based adjuvant QS-21 induced a high magnitude of polyfunctional antigen-specific T cell responses, significantly higher than those observed after dose-matched IM injection [42]. When comparing to unadjuvanted HexaPro HD-MAP vaccination in mice, we saw much lower cellular responses when QS-21 was excluded, although only one cytokine was measured via ELISpot in this work [31]. These findings suggest that the inclusion of adjuvants may be necessary to elicit meaningful T cell responses to HexaPro delivered via the HD-MAP. In contrast, prior clinical studies utilising the HD-MAP to deliver an influenza vaccine demonstrated robust increases in polyfunctional CD4^+^ T cells [4]. This vaccine is also a protein-based vaccine, so perhaps vaccine-intrinsic factors influence the T cell responses after HD-MAP vaccination.

In summary, we demonstrate that HexaPro delivered via the HD-MAP is safe and immunogenic, inducing strong IgG responses that can neutralise both homologous and heterologous viral strains. This makes the HD-MAP platform a promising alternative for SARS-CoV-2 and COVID-19 vaccination.

This study has several limitations. First, as the primary goal of the study was to provide an initial demonstration that HexaPro delivered via the HD-MAP was safe, there was no registered HexaPro vaccine and access to registered vaccines was limited during the period the study was conducted. Therefore, it was not feasible to perform a comparative study comparing the HD-MAP with a needle-delivered comparator or registered SARS-CoV-2 vaccine. Furthermore, there was no adjuvant-free subunit spike vaccine on the market and, when this trial was conducted, access to commercial vaccines was restricted in Australia. Similarly, due to timing of the trial, enrolling naïve individuals was not possible; therefore, we conducted a fourth-dose booster study based on the recommendations at the time by the Australian Government. Participants had received Comirnaty^®^ (Pfizer-BioNTech), Spikevax (Moderna Inc.), or Vaxzevria (AstraZeneca), but the third dose was either Comirnaty or Spikevax. While there were several limitations, the phase 1 trial successfully met its primary goal of evaluating safety of the HexaPro vaccine delivered via the HD-MAP.

## 5. Conclusion

In conclusion, here we report the first-in-human evaluation of the HexaPro SARS-CoV-2 vaccine delivered via an MAP delivery platform. This study demonstrates that the platform is safe, well tolerated, and capable of inducing broad neutralising antibody responses against diverse SARS-CoV-2 variants. The thermostablity observed over 12 months highlights the potential of the HD-MAP technology to reduce the burden on the cold chain and expand vaccine access in low- and middle-income countries. Overall, these findings provide a foundation for advancing the HexaPro HD-MAP and support the broader promise of MAP technologies to improve global vaccine equity.

## Figures and Tables

**Table 1:** Participant details and characteristics.

	Group 1: 0 µg (n = 14)	Group 2: 15 µg (n = 15)	Group 3: 45 µg (n = 15)
**Age (years)**			
**Mean (SD)**	34.6 (11.12)	35.5 (10.71)	32.6 (8.8)
**Range**	19–50	20–49	19–46
**Sex, n (%)**			
**Female**	10 (71.4)	6 (40.0)	11 (73.3)
**Male**	4 (28.6)	9 (60.0)	4 (26.7)
**BMI (kg/m^2^)**			
**Mean (SD)**	24.02 (3.276)	24.65 (3.3)	25.51 (3.2)
**Range**	19.9–31.1	18.3–30.5	20.5–31.1
**Race, number (%)**			
**Aboriginal or Torres Strait Islander**	0 (0)	0 (0)	0 (0)
**Asian**	1 (7.1)	0 (0)	2 (13.3)
**Caucasian**	13 (92.9)	15 (100)	13 (86.7)

**Table 2:** Treatment-emergent adverse events.

		Group 1: 0 µg n = 14 n (%) [e]	Group 2: 15 µg n = 15 n (%) [e]	Group 3: 45 µg n = 15 n (%) [e]	Total n = 44 n (%) [e]
Any		11 (78.6) [26]	12 (80.0) [34]	13 (86.7) [38]	36 (81.8) [98]
Blood and lymphatic system disorders		1 (7.1) [1]	1 (6.7) [1]	1 (6.7) [1]	3 (6.8) [3]
	Lymphadenopathy	1 (7.1) [1]	0 (0.0)	0 (0.0)	1 (2.3) [1]
	Neutrophilia	0 (0.0)	1 (6.7) [1]	0 (0.0)	1 (2.3) [1]
	Thrombocytosis	0 (0.0)	0 (0.0)	1 (6.7) [1]	1 (2.3) [1]
Gastrointestinal disorders		1 (7.1) [1]	3 (20.0) [4]	2 (13.3) [4]	6 (13.6) [9]
	Abdominal pain lower	0 (0.0)	0 (0.0)	1 (6.7) [1]	1 (2.3) [1]
	Diarrhoea	0 (0.0)	0 (0.0)	1 (6.7) [1]	1 (2.3) [1]
	Nausea	1 (7.1) [1]	2 (13.3) [2]	1 (6.7) [1]	4 (9.1) [4]
	Toothache	0 (0.0)	1 (6.7) [1]	0 (0.0)	1 (2.3) [1]
	Vomiting	0 (0.0)	1 (6.7) [1]	1 (6.7) [1]	2 (4.5) [2]
General disorders and administration site conditions		3 (21.4) [3]	6 (40.0) [9]	9 (60.0) [13]	18 (40.9) [25]
	Fatigue	2 (14.3) [2]	1 (6.7) [1]	1 (6.7) [1]	4 (9.1) [4]
	Injection site discolouration	1 (7.1) [1]	0 (0.0)	0 (0.0)	1 (2.3) [1]
	Injection site erythema	0 (0.0)	3 (20.0) [3]	1 (6.7) [1]	4 (9.1) [4]
	Injection site oedema	0 (0.0)	1 (6.7) [1]	0 (0.0)	1 (2.3) [1]
	Injection site pain	0 (0.0)	0 (0.0)	2 (13.3) [2]	2 (4.5) [2]
	Injection site pruritus	0 (0.0)	2 (13.3) [3]	6 (40.0) [7]	8 (18.2) [10]
	Injection site swelling	0 (0.0)	1 (6.7) [1]	1 (6.7) [1]	2 (4.5) [2]
	Malaise	0 (0.0)	0 (0.0)	1 (6.7) [1]	1 (2.3) [1]
Infections and infestations		5 (35.7) [6]	2 (13.3) [3]	2 (13.3) [2]	9 (20.5) [11]
	COVID-19	4 (28.6) [4]	1 (6.7) [1]	0 (0.0)	5 (11.4) [5]
	Influenza	0 (0.0)	1 (6.7) [1]	0 (0.0)	1 (2.3) [1]
	Rhinitis	1 (7.1) [1]	0 (0.0)	0 (0.0)	1 (2.3) [1]
	Upper respiratory tract infection	1 (7.1) [1]	0 (0.0)	1 (6.7) [1]	2 (4.5) [2]
	Viral pharyngitis	0 (0.0)	0 (0.0)	1 (6.7) [1]	1 (2.3) [1]
	Viral upper respiratory tract infection	0 (0.0)	1 (6.7) [1]	0 (0.0)	1 (2.3) [1]
Injury, poisoning, and procedural complications		2 (14.3) [2]	2 (13.3) [2]	1 (6.7) [1]	5 (11.4) [5]
	Back injury	1 (7.1) [1]	0 (0.0)	0 (0.0)	1 (2.3) [1]
	Ligament sprain	0 (0.0)	1 (6.7) [1]	0 (0.0)	1 (2.3) [1]
	Limb injury	1 (7.1) [1]	0 (0.0)	0 (0.0)	1 (2.3) [1]
	Muscle strain	0 (0.0)	1 (6.7) [1]	0 (0.0)	1 (2.3) [1]
	Thermal burn	0 (0.0)	0 (0.0)	1 (6.7) [1]	1 (2.3) [1]
Musculoskeletal and connective tissue disorders		4 (28.6) [6]	2 (13.3) [3]	3 (20.0) [3]	9 (20.5) [12]
	Arthralgia	0 (0.0)	1 (6.7) [1]	1 (6.7) [1]	2 (4.5) [2]
	Back pain	1 (7.1) [1]	0 (0.0)	0 (0.0)	1 (2.3) [1]
	Musculoskeletal stiffness	1 (7.1) [1]	0 (0.0)	1 (6.7) [1]	2 (4.5) [2]
	Myalgia	2 (14.3) [2]	2 (13.3) [2]	1 (6.7) [1]	5 (11.4) [5]
	Neck pain	1 (7.1) [1]	0 (0.0)	0 (0.0)	1 (2.3) [1]
	Pain in extremity	1 (7.1) [1]	0 (0.0)	0 (0.0)	1 (2.3) [1]
Nervous system disorders		5 (35.7) [6]	6 (40.0) [8]	6 (40.0) [10]	17 (38.6) [24]
	Dizziness	0 (0.0)	0 (0.0)	1 (6.7) [1]	1 (2.3) [1]
	Headache	5 (35.7) [6]	5 (33.3) [7]	4 (26.7) [8]	14 (31.8) [21]
	Lethargy	0 (0.0)	1 (6.7) [1]	0 (0.0)	1 (2.3) [1]
	Migraine	0 (0.0)	0 (0.0)	1 (6.7) [1]	1 (2.3) [1]
Respiratory, thoracic, and mediastinal disorders		1 (7.1) [1]	4 (26.7) [4]	2 (13.3) [2]	7 (15.9) [7]
	Cough	1 (7.1) [1]	0 (0.0)	0 (0.0)	1 (2.3) [1]
	Nasal congestion	0 (0.0)	1 (6.7) [1]	0 (0.0)	1 (2.3) [1]
	Oropharyngeal pain	0 (0.0)	2 (13.3) [2]	1 (6.7) [1]	3 (6.8) [3]
	Rhinorrhoea	0 (0.0)	1 (6.7) [1]	0 (0.0)	1 (2.3) [1]
	Sneezing	0 (0.0)	0 (0.0)	1 (6.7) [1]	1 (2.3) [1]
Skin and subcutaneous tissue disorders		0	0	2 (13.3) [2]	2 (4.5) [2]
	Night sweats	0 (0.0)	0 (0.0)	1 (6.7) [1]	1 (2.3) [1]
	Urticaria	0 (0.0)	0 (0.0)	1 (6.7) [1]	1 (2.3) [1]

Results are presented as the number of subjects with the event: n, the proportion of subjects with the event: (%), and the number of events: [e]. TEAE, treatment-emergent adverse event.

**Table 3:** Localised and systemic study treatment-related treatment-emergent adverse events.

		Group 1: 0 µg n = 14 n (%) [e]	Group 2: 15 µg n = 15 n (%) [e]	Group 3: 45 µg n = 15 n (%) [e]	Total n = 44 n (%) [e]
**Localised**					
Any		2 (14.3) [2]	6 (40.0) [9]	9 (60.0) [12]	17 (38.6) [23]
General disorders and administration site conditions		1 (7.1) [1]	6 (40.0) [8]	8 (53.3) [11]	15 (34.1) [20]
	Injection site discolouration	1 (7.1) [1]	0 (0.0)	0 (0.0)	1 (2.3) [1]
	Injection site erythema	0 (0.0)	3 (20.0) [3]	1 (6.7) [1]	4 (9.1) [4]
	Injection site oedema	0 (0.0)	1 (6.7) [1]	0 (0.0)	1 (2.3) [1]
	Injection site pain	0 (0.0)	0 (0.0)	2 (13.3) [2]	2 (4.5) [2]
	Injection site pruritus	0 (0.0)	2 (13.3) [3]	6 (40.0) [7]	8 (18.2) [10]
	Injection site swelling	0 (0.0)	1 (6.7) [1]	1 (6.7) [1]	2 (4.5) [2]
Musculoskeletal and connective tissue disorders		1 (7.1) [1]	1 (6.7) [1]	1 (6.7) [1]	3 (6.8) [3]
	Arthralgia	0 (0.0)	0 (0.0)	1 (6.7) [1]	1 (2.3) [1]
	Myalgia	0 (0.0)	1 (6.7) [1]	0 (0.0)	1 (2.3) [1]
	Pain in extremity	1 (7.1) [1]	0 (0.0)	0 (0.0)	1 (2.3) [1]
**Systemic**					
Any		4 (28.6) [7]	5 (33.3) [8]	5 (33.3) [12]	14 (31.8) [27]
Gastrointestinal disorders		0	1 (6.7) [1]	1 (6.7) [3]	2 (4.5) [4]
	Diarrhoea	0 (0.0)	0 (0.0)	1 (6.7) [1]	1 (2.3) [1]
	Nausea	0 (0.0)	1 (6.7) [1]	1 (6.7) [1]	2 (4.5) [2]
	Vomiting	0 (0.0)	0 (0.0)	1 (6.7) [1]	1 (2.3) [1]
General disorders and administration site conditions		2 (14.3) [2]	1 (6.7) [1]	2 (13.3) [2]	5 (11.4) [5]
	Fatigue	2 (14.3) [2]	1 (6.7) [1]	1 (6.7) [1]	4 (9.1) [4]
	Malaise	0 (0.0)	0 (0.0)	1 (6.7) [1]	1 (2.3) [1]
Musculoskeletal and connective tissue disorders		2 (14.3) [3]	1 (6.7) [1]	1 (6.7) [1]	4 (9.1) [5]
	Musculoskeletal stiffness	1 (7.1) [1]	0 (0.0)	0 (0.0)	1 (2.3) [1]
	Myalgia	2 (14.3) [2]	1 (6.7) [1]	1 (6.7) [1]	4 (9.1) [4]
Nervous system disorders		2 (14.3) [2]	4 (26.7) [5]	2 (13.3) [3]	8 (18.2) [10]
	Headache	2 (14.3) [2]	3 (20.0) [4]	2 (13.3) [3]	7 (15.9) [9]
	Lethargy	0 (0.0)	1 (6.7) [1]	0 (0.0)	1 (2.3) [1]
Respiratory, thoracic, and mediastinal disorders		0	0	1 (6.7) [1]	1 (2.3) [1]
	Oropharyngeal pain	0 (0.0)	0 (0.0)	1 (6.7) [1]	1 (2.3) [1]
Skin and subcutaneous tissue disorders		0	0	2 (13.3) [2]	2 (4.5) [2]
	Night sweats	0 (0.0)	0 (0.0)	1 (6.7) [1]	1 (2.3) [1]
	Urticaria	0 (0.0)	0 (0.0)	1 (6.7) [1]	1 (2.3) [1]

Results are presented as the number of subjects with the event: n, the proportion of subjects with the event: (%), and the number of events: [e]. TEAE, treatment-emergent adverse event.

## Data Availability

All data reported in this paper is available in this article.

## Supplementary Materials

The following supporting information can be downloaded at: https://www.mdpi.com/article/10.3390/vaccines14010028/s1, Figure S1. SARS-CoV-2 nucleocapsid-specific IgG titers in serum; Figure S2. SARS-CoV-2 nucleocapsid-specific IgA titers in saliva samples; Figure S3. Relationship between incoming and later timepoint IgG titer; Figure S4. SARS-CoV-2 spike- and RBD-specific IgA responses in the saliva; Table S1. Mean IgG titer fold change of serum from patients measured against various SARS-CoV-2 spike proteins.

## Abbreviations

The following abbreviations are used in this manuscript:

AU	Assay units
BCA	Bicinchoninic acid
CMC	Carboxymethyl cellulose
cT_FH_	Circulating T follicular helper cells
CTN	Clinical trials notification
ELISA	Enzyme-linked immunosorbent assay
GCP	Good clinical practice
HD-MAP	High-density microarray patch
HIC	High income country
MAP	Microarray patch
NHMRC	National Health and Medical Research Council
RF10	RPMI-1640 with 10% fetal calf serum, penicillin and streptomycin
rHSA	Recombinant human serum albumin
TEAE	Treatment emergent adverse event
TGA	Therapeutic Goods Administration
TMB	3,3′,5,5′-tetramethylbenzidine
