# Supplementary material for: Booster Immunisation with Skin-Patch-Delivered Unadjuvanted SARS-CoV-2 Spike Protein Vaccine Is Safe and Immunogenic in Healthy Adults
Source: Vaccines (Basel). 2025 Dec 25;14(1):28. doi: 10.3390/vaccines14010028 (PMC12846601; doi:10.3390/vaccines14010028)
Supplement: Supplementary file 1 [file vaccines-14-00028-s001.zip › vaccines-3918330-supplementary.pdf]

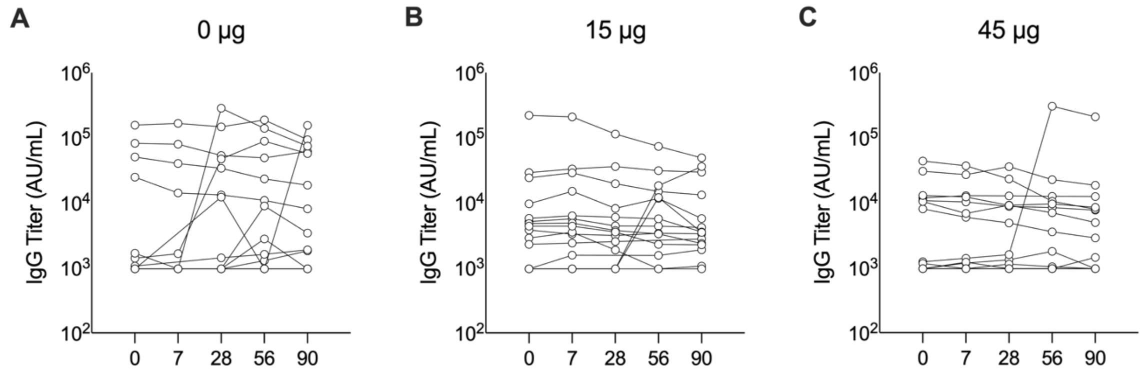

Figure. S1. SARS-CoV-2 nucleocapsid-specific IgG titers in serum.

Serum collected from subjects from each group were analyzed for their nucleocapsid-specific IgG titer **(A)** 0 μg group, **(B)** 15 μg group and **(C)** 45 μg group. Each line and dot represents an individual subject. AU, antibody units.

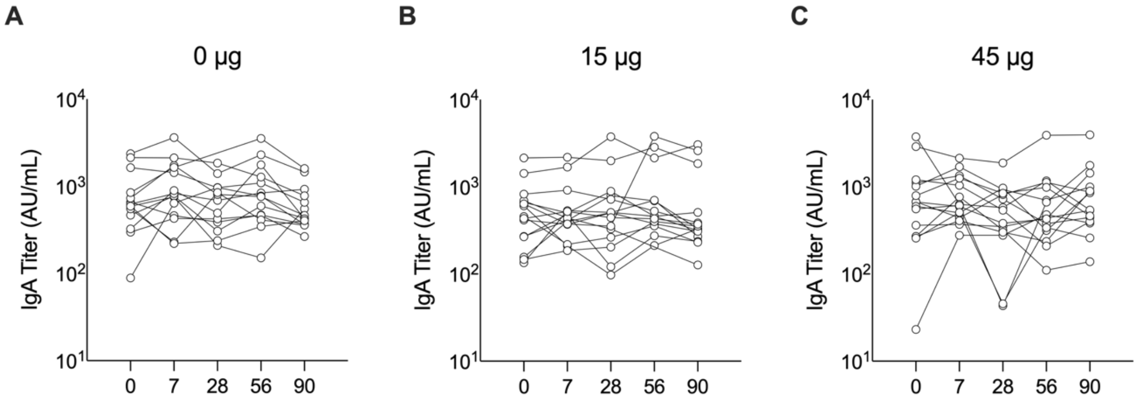

Figure S2. SARS-CoV-2 nucleocapsid-specific IgA titers in saliva samples.
Saliva collected from subjects from each group were analyzed for their nucleocapsid-specific IgA titer **(A)** 0 μg group, **(B)** 15 μg group and **(C)** 45 μg group. Each line and dot represents an individual subject. AU, antibody units.

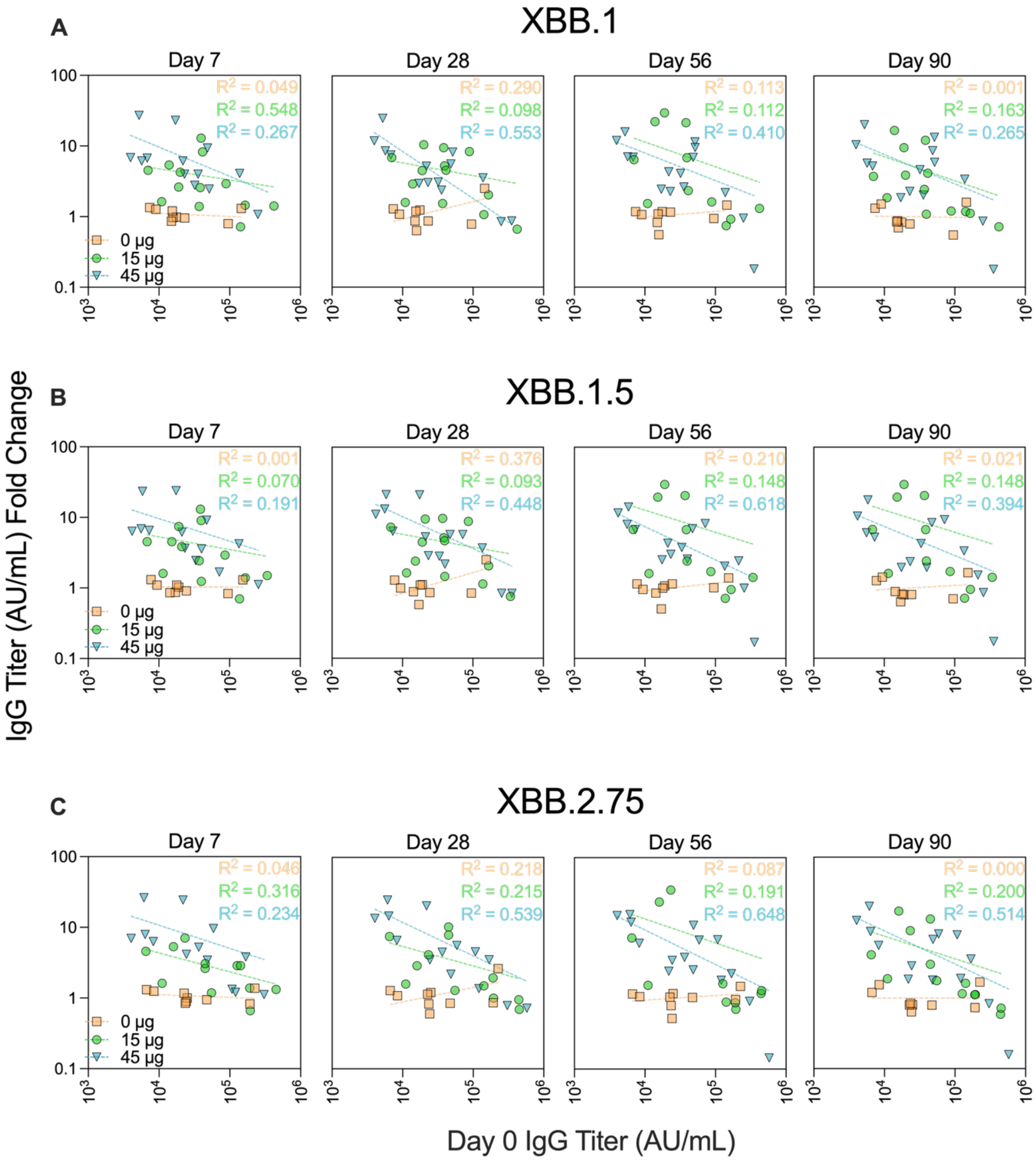

Figure S3. Relationship between incoming and later timepoint IgG titer.

The relationship between the spike-specific IgG titer prior to vaccination (day 0) and the timepoints taken after vaccination for **(A)** BB.1, **(B)** XBB.1.5 and **(C)** XBB.2.75 spike protein. AU, antibody units.

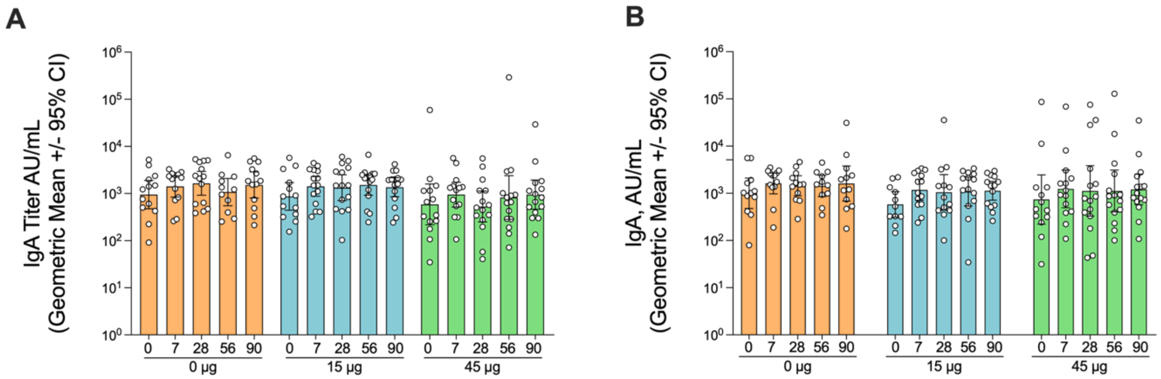

Figure S4. SARS-CoV-2 spike- and RBD-specific IgA responses in the saliva.

Saliva was collected on days 0, 7, 28, 56 and 90 and analyzed for IgA titre against (A) wildtype spike protein or (B) the receptor binding domain (RBD) of the wildtype spike protein. Data presented as the geometric mean with error bars showing the 95% confidence intervals. AU, antibody units.

Table S1.
Mean IgG titer fold change of serum from patients measured against various SARS-CoV-2 spike proteins.

		Day			
	**Group**	**7**	**28**	**56**	**90**
**WT**	**0**	1.01	1.19	1.67	1.67
	**15**	2.77	4.18	4.42	3.76
	**30**	5.38	5.48	4.83	4.32
**XBB.1**	**0**	1.07	2.04	3.24	4.26
	**15**	3.68	4.32	8.44	4.62
	**30**	7.99	6.37	5.99	6.10
**XBB.1.5**	**0**	1.05	1.95	3.16	4.06
	**15**	4.00	4.28	8.14	8.14
	**30**	7.63	7.36	5.18	5.28
**XBB.2.75**	**0**	1.05	1.80	2.69	4.91
	**15**	2.70	3.27	7.16	4.37
	**30**	7.82	7.69	6.07	5.94
